# Supplementary material for: Biomechanical, Microstructural and Material Properties of Tendon and Bone in the Young Oim Mice Model of Osteogenesis Imperfecta
Source: Int J Mol Sci. 2022 Sep 1;23(17):9928. doi: 10.3390/ijms23179928 (PMC9456454; doi:10.3390/ijms23179928)
Supplement: Supplementary file 1 [file ijms-23-09928-s001.zip › ijms-1841243-supplementary.pdf]

Supplementary Table : Clinical manifestations of osteogenesis imperfecta according to Sillence classification. Only types caused by collagen mutation are listed here.

**Types**	**Morbidity**	**Clinical manifestations**	
		Skeletal	Extra-skeletal
I	Mild	Short or normal stature, few fracture	Blue sclera, muscle weakness, mild joint laxity
II	Perinatal lethality	Multiple ribs and long bones fractures, severe deformities	Severe pulmonary insufficiency
III	Severe	Short stature, severe scoliosis, fracture and deformities, triangle-shaped face	Blue sclera, dentinogenesis imperfecta, hearing loss, joint laxity
IV	Moderate	Fracture and deformities	Blue sclera, dentinogenesis imperfecta
